# Thyrotoxic Paralysis in a Hispanic Woman: An Unusual Presentation of a Neurological Emergency

**DOI:** 10.7759/cureus.104037

**Published:** 2026-02-21

**Authors:** Anchu A Vincent, Era Allili, Joseph Abraham, Uloma E Obi, Manali Patel, Krupali Acharya

**Affiliations:** 1 Internal Medicine, Harlingen Medical Center, Harlingen, USA; 2 Medicine, Knapp Medical Center, Weslaco, USA

**Keywords:** hyperthyroidism, muscle weakness, normokalemic periodic paralysis, thyrotoxicosis, thyrotoxic periodic paralysis

## Abstract

Thyrotoxic periodic paralysis (TPP) is an uncommon but potentially life-threatening complication of hyperthyroidism characterized by sudden, reversible muscle weakness due to an intracellular potassium shift. While hypokalemia is a typical feature, normokalemic presentations are exceedingly rare. We report a 46-year-old Hispanic woman with poorly controlled hyperthyroidism who presented with acute bilateral lower-extremity weakness and areflexia. The initial potassium level was within the normal range. The patient was clinically and biochemically thyrotoxic. She was treated with methimazole, dexamethasone, propranolol, lisinopril, and cautious electrolyte replacement, resulting in progressive improvement and complete recovery of motor strength. Although TPP predominantly affects Asian males, this case illustrates that it can occur in other ethnicities and in females. Acute flaccid paralysis in hyperthyroid patients, even with normal serum potassium, should prompt suspicion for TPP to avoid unnecessary neurologic workups and delays in therapy. Early recognition and prompt administration of β-blockers and antithyroid therapy are critical to prevent complications and recurrence.

## Introduction

Thyrotoxic periodic paralysis (TPP) is a rare but potentially life-threatening neuromuscular emergency resulting from thyrotoxicosis. It is characterized by transient flaccid paralysis and areflexia, typically involving the lower limbs, caused by intracellular potassium redistribution, resulting in hypokalemic paralysis [1]. Although well recognized among the Asian population - particularly among males of Chinese, Japanese, and Filipino descent - the condition is increasingly being identified in other ethnic groups, including Hispanics, Caucasians, and African Americans [2]. The incidence of TPP among hyperthyroid patients in North America is estimated to be less than 0.1% [1]. While hypokalemia is a diagnostic hallmark, a few cases with normokalemia have been reported, expanding the pathophysiologic spectrum of the disease.

Because acute flaccid paralysis has a broad differential - ranging from Guillain-Barré syndrome to hypokalemic paralysis or spinal cord pathology - early recognition of TPP is essential to avoid unnecessary diagnostic testing and prevent potential respiratory compromise. This report highlights a rare case of normokalemic TPP in a Hispanic female with uncontrolled hyperthyroidism, emphasizing that TPP can occur across diverse populations and potassium levels, and that prompt recognition with appropriate β-blocker and antithyroid therapy can lead to full recovery.

## Case presentation

A 46-year-old Hispanic woman presented to the emergency department in September 2024 with the sudden onset of bilateral lower-extremity weakness accompanied by muscle soreness. The weakness developed progressively over several hours, eventually making it difficult for her to stand or walk. She denied any sensory disturbances, such as numbness or paresthesia, as well as headache, bowel or bladder incontinence, recent strenuous exercise, or prolonged fasting.

Her medical history was significant for hypertension, chronic low back pain, and chronic hyperthyroidism, with documented non-compliance to methimazole therapy. She was an active smoker and reported occasional alcohol consumption. Notably, she described an unintentional weight loss exceeding 100 pounds over the past several years. At the time of presentation, she was not taking any prescribed medications.

On examination, her temperature was 98.1 °F, with a heart rate of 110 beats per minute and blood pressure of 169/88 mm Hg. The neurological examination revealed flaccid paralysis with motor strength graded 1/5 in both lower extremities, accompanied by areflexia but preserved sensation. Cranial nerve function and upper limb strength were intact, and no cerebellar signs or sensory level were identified.

Pertinent laboratory results at the time of admission are shown in Table 1.

**Table 1:** Initial Laboratory Results on Admission TSH: Thyroid-stimulating hormone

Test	Result	Reference ranges
Sodium	141 mmol/L	135-145 mmol/L
Potassium	3.6 mmol/L	3.5-5.0 mmol/L
Magnesium	1.6 mg/dL	1.7-2.4 mg/dL
TSH	<0.005 µIU/mL	0.4-4.5 µIU/mL
Free T3	569 ng/dL	80-180 ng/dL
Free T4	6.14 ng/dL	0.8-1.8 ng/dL

**Figure 1:**
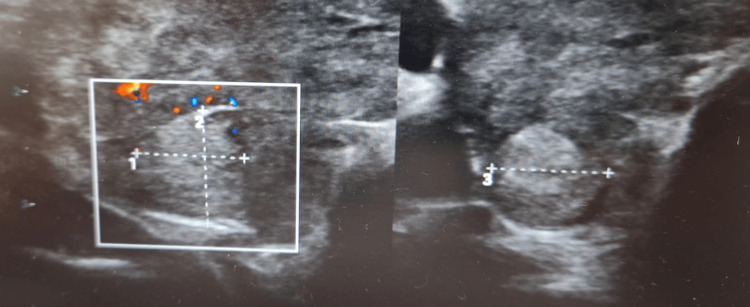
Ultrasound Thyroid Showing a 1 cm Nodule

Urine culture grew *Escherichia coli*, for which the patient was started on appropriate antibiotic therapy. Thyroid ultrasound (Figure 1) revealed a 1-cm hypoechoic nodule in the right lower pole, consistent with a benign-appearing lesion.

MRI of the lumbar spine demonstrated moderate-to-severe L5-S1 canal stenosis secondary to anterolisthesis; however, neurosurgical evaluation determined the finding to be chronic, and conservative management with a lumbar brace was recommended.

During hospitalization, she was treated with methimazole, dexamethasone, and propranolol for thyrotoxicosis (Table 2). Electrolyte replacement with potassium and magnesium was initiated due to laboratory evidence of mild hypomagnesemia and borderline potassium levels. Her muscle strength gradually improved with therapy, and by hospital day nine, she was able to ambulate with minimal assistance. The patient's hospital course is summarized in Table 2.

**Table 2:** Timeline of Hospital Management and Clinical Recovery

Hospital day	Key findings	Potassium level (mmol/L)	Lower limb power	Interventions
1	Lower extremity weakness and areflexia	3.6	1/5	Started methimazole 5 mg TID, dexamethasone 6 mg daily, propranolol 10 mg QID, lisinopril 20 mg daily
3	Started tolerating physical therapy	3.5	2/5	Urine culture + *Escherichia coli* → Zosyn started
6	Improving lower extremity strength	3.4	3/5	Potassium supplementation added
9	Ambulating with minimal assistance	3.6	4/5	Discharged home

The patient was discharged home after clinical improvement. She was subsequently lost to outpatient follow-up and was nonadherent to prescribed medications. Two months later, she was readmitted with atrial flutter in the setting of persistent hyperthyroidism. During this admission, she denied any recurrent episodes of muscle weakness or paralysis since prior hospitalization. She was started on methimazole 10 mg three times daily, propranolol 60 mg daily, and apixaban 5 mg twice daily and was counseled extensively on medication adherence.

## Discussion

TPP is a reversible neuromuscular emergency caused by intracellular potassium shift into skeletal muscle in the setting of thyrotoxicosis [3]. Unlike most thyroid disorders, TPP classically affects Asian males between 20 and 40 years of age[1] and is typically associated with marked hypokalemia. Normokalemic presentations of TPP have been reported and represent an atypical clinical variant[4].

Our patient, a middle-aged Hispanic woman with normal potassium levels, presented with acute reversible lower extremity flaccid paralysis in the setting of overt thyrotoxicosis and responded rapidly to treatment of thyrotoxicosis. These atypical features complicated the diagnostic process. A comparison between our patient's presentation and classic TPP features is summarized in Table 3.

**Table 3:** Comparison of Patient Features With Classical TPP Characteristics TPP: Thyrotoxic periodic paralysis

Feature	Current Case	Typical TPP
Demographics	Hispanic female, 46 y	Asian male, 20-40 y
Family history	Negative	Positive or negative
Serum potassium	3.4-3.6 mmol/L	< 3.0 mmol/L
Reflexes	Absent	Decreased/absent
Occurrence	First episode	Recurrent episodes
Thyroid state	Overt thyrotoxicosis	Subtle or overt thyrotoxicosis
Triggers	Possible carbohydrate and alcohol	High-carb meals, exercise, stress
Recovery	Complete after euthyroid correction	Complete after euthyroid correction

Normokalemic paralysis is uncommon and may result from transient potassium shifts before blood sampling, magnesium deficiency, direct thyrotoxic myopathy, or genetic predisposition due to ion-channel mutations [4,5]. Mutations in KCNJ2 and KCNJ18, encoding skeletal muscle inward-rectifying potassium channels (Kir2.1 and Kir2.6), have been linked to increased susceptibility and may explain atypical presentations despite normal serum potassium levels [5]. Because hypokalemia is often considered essential for diagnosis, alternative etiologies of paralysis, such as Guillain-Barré syndrome, spinal cord pathologies can be pursued. In our case, although the lumbar spine showed moderate lumbar stenosis, neurosurgery deemed it to be of a chronic nature and recommended conservative management.

The absence of sensory deficits, preservation of cranial nerve functions, and rapid reversibility of weakness following beta -blocker and methimazole therapy strongly supported the diagnosis of normokalemic TPP variant rather than a primary neurologic disorder [4]. Our patient was readmitted two months later, with atrial flutter secondary to uncontrolled hyperthyroidism after medication nonadherence. Notably, she reported no recurrence of paralysis during that interval. This further supports the episodic and trigger-dependent nature of TPP and highlights that maintenance of a euthyroid state is essential.

The cornerstone of management involves suppression of thyroid hormone-induced adrenergic activity with nonselective β-blockers such as propranolol, along with initiation of antithyroid therapy (methimazole or propylthiouracil)[6]. Cautious correction of electrolyte abnormalities, particularly potassium and magnesium, is recommended; overcorrection should be avoided, as paralysis typically resolves with redistribution rather than true potassium depletion [7]. Definitive therapy requires restoration and maintenance of a euthyroid state to prevent recurrence and complications[8,9].

## Conclusions

This case underscores the diagnostic challenge of normokalemic TPP in non-Asian populations. The presence of acute, reversible flaccid paralysis in a hyperthyroid patient - even with normal potassium - should prompt consideration of TPP. Recognizing such atypical presentations can prevent unnecessary neurological investigations and avert potential complications such as respiratory compromise or arrhythmia.

Prompt initiation of β-adrenergic blockade (e.g., propranolol) and antithyroid therapy (e.g., methimazole or propylthiouracil) remains the cornerstone of management, with potassium replacement used cautiously to avoid rebound hyperkalemia once intracellular potassium shifts normalize. Definitive prevention requires achievement of the euthyroid state, which eliminates recurrence risk.

## References

[1] Lin SH (2005). Thyrotoxic periodic paralysis. Mayo Clin Proc.

[2] Kung AW (2006). Thyrotoxic periodic paralysis: a diagnostic challenge. J Clin Endocrinol Metab.

[3] Ryan DP, da Silva MR, Soong TW (2010). Mutations in potassium channel Kir2.6 cause susceptibility to thyrotoxic hypokalemic periodic paralysis. Cell.

[4] Singh B, Singh B, Sacoto D, Lee S (2024). Thyrotoxic normokalemic periodic paralysis (TNPP), navigating the benefits of early therapeutic strategies. Endocr Pract.

[5] Cheng CJ, Kuo E, Huang CL (2013). Extracellular potassium homeostasis: insights from hypokalemic periodic paralysis. Semin Nephrol.

[6] McFadzean AJ, Yeung R (1967). Periodic paralysis complicating thyrotoxicosis in Chinese. Br Med J.

[7] Bernard JD, Larson MA, Norris FH Jr (1972). Thyrotoxic periodic paralysis in Californians of Mexican and Filipino ancestry. Calif Med.

[8] Lin SH, Huang CL (2012). Mechanism of thyrotoxic periodic paralysis. J Am Soc Nephrol.

[9] Ober KP (1992). Thyrotoxic periodic paralysis in the United States. Report of 7 cases and review of the literature. Medicine (Baltimore).

